# The Health Benefits and Challenges of Exercise Training in Persons Living with Schizophrenia: A Pilot Study

**DOI:** 10.3390/brainsci3020821

**Published:** 2013-05-24

**Authors:** Shannon S. D. Bredin, Darren E. R. Warburton, Donna J. Lang

**Affiliations:** 1 Cognitive and Functional Learning Laboratory, University of British Columbia, Vancouver V6T 1Z1, Canada; E-Mails: shannon.bredin@ubc.ca (S.S.D.B.); darren.warburton@ubc.ca (D.E.R.W.); 2 Physical Activity Promotion and Chronic Disease Prevention Unit, University of British Columbia, Vancouver V6T 1Z1, Canada; 3 Cardiovascular Physiology and Rehabilitation Laboratory, University of British Columbia, Vancouver V6T 1Z1, Canada; 4 International Collaboration on Repair Discoveries, University of British Columbia, Vancouver V5Z 1M9, Canada; 5 Department of Radiology, University of British Columbia, Vancouver V6T 1Z1, Canada

**Keywords:** schizophrenia, exercise, cardio-metabolic disease, cognitive function, functional limitations

## Abstract

Background: In addition to the hallmark cognitive and functional impairments mounting evidence indicates that schizophrenia is also associated with an increased risk for the development of secondary complications, in particular cardio-metabolic disease. This is thought to be the result of various factors including physical inactivity and the metabolic side effects of psychotropic medications. Therefore, non-pharmacological approaches to improving brain health, physical health, and overall well-being have been promoted increasingly. Methods: We report on the health-related physical fitness (body composition, blood pressure, heart rate, and aerobic fitness) and lipid profile of persons living with schizophrenia and effective means to address the challenges of exercise training in this population. Results: There was a markedly increased risk for cardio-metabolic disease in 13 persons living with schizophrenia (Age = 31 ± 7 years) including low aerobic fitness (76% ± 34% of predicted), reduced HDL (60% of cohort), elevated resting heart rate (80% of cohort), hypertension (40% of cohort), overweight and obesity (69% of cohort), and abdominal obesity (54% of cohort). Individualized exercise prescription (3 times/week) was well tolerated, with no incidence of adverse exercise-related events. The exercise adherence rate was 81% ± 21% (Range 48%–100%), and 69% of the participants were able to complete the entire exercise training program. Exercise training resulted in clinically important changes in physical activity, aerobic fitness, exercise tolerance, blood pressure, and body composition. Conclusion: Persons living with schizophrenia appear to be at an increased risk for cardio-metabolic disease. An individualized exercise program has shown early promise for the treatment of schizophrenia and the various cognitive, functional, and physiological impairments that ultimately affect health and well-being.

## 1. Introduction

Schizophrenia is a severe mental illness that generally presents in early adulthood (affecting approximately 1% of the Canadian population) [1,2]. This condition is characterized by acute psychotic episodes and marked social and occupational dysfunction. The economic burden associated with schizophrenia is significant approximating $6.85 billion in 2004 [3]. 

The inability to carry out normal social and/or occupational activities is associated with marked cognitive impairments, particularly related to memory and attention [4,5]. Impaired memory and small hippocampal volumes [6,7] are amongst the most common and consistent findings in persons living with schizophrenia, and these findings appear to be interrelated [8,9]. Current antipsychotic medications reduce the symptoms of psychosis (such as delusions and hallucinations); however, these medications may have limited effects upon the cognitive and functional impairments that are the hallmarks of schizophrenia [10,11]. Moreover, these medications have various side effects that place patients with schizophrenia at increased risk for the development of other chronic medical conditions. 

Persons living with schizophrenia have an increased risk for developing the metabolic syndrome, type 2 diabetes, and cardiovascular disease [12,13]. The prevalence of overweight, obesity, and hyperglycemia is markedly higher in persons living with mental illness in comparison to the general population. Approximately 60%–70% of patients living with schizophrenia are overweight or obese, with 30%–40% being obese [14]. These patients have approximately 16% greater prevalence of the metabolic syndrome [15], and are at a 2- to 3-fold increased risk of developing and dying from heart disease and diabetes [15,16,17,18,19] that is thought to correspond to a 10–25 year reduction in life expectancy [20]. Recent evidence indicates a high risk of fatal cardiovascular events over a ten-year period in patients with schizophrenia [21]. Increased cardio-metabolic risk has been associated with a variety of factors including reduced physical activity, poor nutrition, poverty, reduced medical care access, and the metabolic side effects of psychotropic medications [12]. In fact, antipsychotic medications (particularly second generation medications such as risperidone, olanzapine, and clozapine) are associated with excessive weight gain, movement disorders, and alterations in glucose (e.g., insulin resistance) and lipid (dsylipidemia) metabolism [22,23,24]. Collectively, patients living with schizophrenia are at a markedly increased risk for developing cardio-metabolic disease and premature mortality [25].

Owing to the cognitive impairment in schizophrenia and the side effects of antipsychotic medications there is a clear need to explore non-pharmacological approaches to improving brain health, physical health, and overall well-being. There is considerable evidence that routine physical activity is associated with a reduced risk for various chronic conditions (including type 2 diabetes and cardiovascular disease) and an improvement in cognitive function [26,27]. 

There is growing interest in the potential health benefits of exercise in persons living with schizophrenia [28]. This increased attention is owing to the multiple health benefits of exercise decreasing the risk for the development of secondary complications and premature mortality, and the potential for exercise to specifically target cognitive deficits and/or hippocampal abnormalities in schizophrenia. In fact, routine physical activity/exercise has been show to decrease the severity of negative symptoms [29,30,31], reduce stress and anxiety [32], improve concentration and attention [33], and reduce depression severity [34] in schizophrenia. A recent systematic review of the literature [28] revealed that there is preliminary evidence from randomized controlled trials that aerobic and strength exercises (and yoga) may reduce state anxiety, psychiatric symptoms, and distress, and improve quality of life and short term memory in schizophrenia. We hypothesize that exercise affects hippocampal integrity explaining (at least in part) the improvements in cognition and the reduction of symptoms in psychosis. 

Despite the clear health benefits of engaging in routine physical activity and exercise training, patients living with schizophrenia are amongst the least likely to engage in regular exercise. Accordingly, our research team has examined the most effective means to evaluate health-related physical fitness (*i.e.*, body composition, blood pressure, heart rate, aerobic fitness) and create individualized exercise prescriptions for persons living with schizophrenia. This present report highlights the successes and challenges of our research to date in developing effective exercise prescriptions for persons living with schizophrenia. This includes highlighting our newly created centre (*i.e.*, the Centre of Excellence in Brain Health and Wellness) that is dedicated to improving the health and well-being of persons living with major mental illness. For the current paper we focus on the heath-related physical fitness and lipid profile of persons living with schizophrenia, and the successes and challenges of exercise training in this population. When beginning the research trial, we anticipated that traditional exercise rehabilitation strategies would need to be modified considerably according to the unique needs of persons living with schizophrenia. We also hypothesized that exercise adherence and participant drop-out (attrition) would be a significant issue that would challenge the effectiveness of this intervention. 

## 2. Methodology

Ethics approval for this study was provided by the University of British Columbia Research Ethics Board in accordance with Tri-council Guidelines. Thirteen patients (7 Males; 6 Females) with schizophrenia enrolled in our exercise trial in the Centre of Excellence in Brain Health and Wellness at the University of British Columbia according to various inclusion and exclusion criteria (Table 1). Two additional participants were approached and declined to participate in the intervention. Participants completed the Physical Activity Readiness Questionnaire for Everyone (PAR-Q+) and electronic Physical Activity Medical Readiness Questionnaire (ePARmed-X+) with the assistance of a qualified exercise professional [35], provided written informed consent, and received physician clearance for participation in the exercise trial. We have previously validated the PAR-Q+ and ePARmed-X+ [36]. There was 100% agreement in those cleared for physical activity participation using both methods. This research was conducted in accordance with the *Declaration of Helsinki*. 

**Table 1:** Inclusion and exclusion criteria for participants living with schizophrenia.

Inclusion Criteria	Exclusion Criteria
Age 21 to 45 years Able to provide written, informed consent in English Patients may be on prescribed medications DSM-IV (Diagnostic and Statistical Manual of Mental Disorders, Fourth Edition) diagnosis of schizophrenia or schizoaffective disorder Normal visual acuity (or normal visual acuity achievable with corrective lenses) Physical ability to engage in a regular exercise program (as determined by their treating physician and the completion of the ePARmed-X+)	A history of developmental disorders (e.g., autism, mental retardation, Down’s Syndrome) A current DSM-IV diagnosis of substance dependence (during prior 120 days, excluding tobacco) Any history of DSM-IV diagnoses (Axis I) for other psychiatric disorders History of angina, heart attack, or transient ischemic attacks Non-independent mobility or limb prostheses A history of organic disorders or severe head trauma (e.g., dementia or head injury leading to loss of consciousness for >5 min) Contraindications for neuroimaging (metal implants, non-removable orthodonic devices, severe claustrophobia, pregnancy, or surgeries within the previous six months) Already enrolled currently in a regular exercise program Currently not stable on medications

The patients were asked to participate in a series of tests before and after exercise training (*i.e.*, baseline, 6 weeks, and 12 weeks). This included the assessment of various markers of health-related physical fitness (including body mass and composition, aerobic fitness, blood pressure, and heart rate) and lipid profile. We also assessed demographics and physical activity behaviours. Program adherence (attendance) was monitored objectively throughout the program via direct recording of attendance at exercise sessions. The total exercise adherence rate was calculated based on the total possible exercise sessions (considering dropout from the study) and the actual exercise sessions attended. Moreover, the dropout rate (attrition) of participants was calculated (considering those that started and did not complete the entire 12 week trial).

### 2.1. Physical Measures

#### 2.1.1. Anthropometry and Body Composition

Body mass (kg) and standing height (cm) were measured according to standard procedures (via calibrated weight and height scales) and body mass index was calculated. Waist circumference was measured using an anthropometric tape according to the recommendations of the National Institutes of Health (*i.e.*, at the top border of the iliac crest) [37]. Body composition was further refined (according to the health-related procedures of Gledhill and Jamnik [38]) via the measurement of skinfolds (triceps, biceps, subscapular, supra iliac, and medial calf). 

#### 2.1.2. Resting Lipid Profile

Fasting (overnight) blood samples were collected by a registered nurse between 7:00 and 9:00 a.m. High density lipoprotein, total cholesterol, and triglycerides were evaluated independently by the Department of Pathology and Laboratory Medicine (Vancouver General Hospital) via respective Flex^®^ Reagent Cartridges on the Dimension Vista^®^ System (Siemens, Healthcare Diagnostics, Newark, Delaware, USA). The low density lipoprotein cholesterol (*i.e.*, low density lipoprotein = cholesterol − high density lipoprotein − (triglycerides/2.2)) and total cholesterol to high density lipoprotein ratio (*i.e.*, cholesterol/high density lipoprotein) were calculated.

#### 2.1.3. Resting Blood Pressure and Heart Rate

Resting blood pressure (sphygmomanometer and stethoscope) and heart rate were recorded in the seated upright posture at each testing time period. Both resting blood pressure and heart rate were classified according to current recommendations for the risk of cardiovascular disease. The risk categories according to resting blood pressure (established by the American Heart Association) are as follows: Normal (systolic blood pressure >120 mmHg and diastolic blood pressure >80 mmHg); Pre-hypertension (systolic blood pressure 120–139 mmHg and/or diastolic blood pressure 80–89 mmHg); Stage I hypertension (systolic blood pressure 140–159 mmHg and/or diastolic blood pressure 90–99 mmHg; Stage II Hypertension (systolic blood pressure 160 mmHg or greater and/or diastolic blood pressure 100 mmHg or higher) [39]. Resting heart rate was categorized into higher risk (resting heart rate >90 bpm) and lower risk according to the recommendations of Cooney and colleagues [40]. 

#### 2.1.4. Aerobic Fitness

We evaluated aerobic fitness using an incremental to maximum exercise test on an electronically braked cycle ergometer. Starting at a power output of 20 W, the protocol increased 15 W/min until the participant reached volitional fatigue. Expired gas and ventilatory parameters were acquired throughout using a calibrated metabolic cart (Ergocard, Medisoft) allowing for the determination of peak aerobic power (VO_2peak_). Heart rate (12-lead ECG) and oxyhaemoglobin saturation (pulse oximetery) were monitored continuously. Ratings of perceived exertion and blood pressure were assessed every 2 min. A qualified exercise professional and the research coordinator supervised all tests. Exercise tests would have been terminated immediately if one or more of the following symptoms occur: (1) chest pain, (2) ST segment depression or elevation of >1 mm beyond baseline (the ST segment refers to the flatline segment of a standard electrocardiogram tracing), (3) a significant decrease in systolic blood pressure during exercise (>10 mmHg), and/or (4) other abnormal ECG responses. None of these symptoms were observed in this trial, and all participants were able to complete the test. Each participant’s VO_2peak_ was expressed as a percentage of predicted according to the formula of Jones [41]:

%Predicted VO_2peak_ = (0.032 × Ht) − (0.024 × Age) + (0.019 × Wt) − (0.49 × Sex) − 3.17

where Ht = height in m, Wt = body mass in kg, and sex is coded 0 for males and 1 for females.

#### 2.1.5. Physical Activity

General physical activity was assessed by a modified Godin Shephard Leisure Time Exercise Questionnaire [42,43]. A modification of the Godin Shephard Leisure Time Questionnaire was made to include the average duration of activity for each respective category. As such, participants were asked to report the average weekly frequency and duration of light (minimal effort, no perspiration), moderate (not exhausting, light perspiration), and vigorous (heart beats rapidly, sweating) physical activities [44]. We calculated the weekly leisure-time activity score according to the suggestions of Godin [45] as: Weekly leisure-time activity score = (9 × Strenuous) + (5 × Moderate) + (3 × Mild). Using the weekly leisure-time activity score we classified the participants into health-related activity categories according to the recommendations of Godin including: (a) Active (≥24 units reflecting substantial health benefits), (b) Moderately Active (14–23 units reflecting some health benefits), and (c) Insufficiently Active (<14 units reflecting less/low health benefits) [45]. 

The physical activity behaviours of the participants were also compared to international recommendations for physical activity (*i.e.*, ≥75 min of vigorous or ≥150 min of moderate aerobic physical activity per week, or an equivalent combination) [46]. Total physical activity minutes were calculated as: Physical Activity Minutes = Moderate Duration + (2 × Vigorous Duration). Participants were categorized as: (a) meeting international physical activity guidelines (≥150 Physical Activity Minutes), (b) insufficiently active (1–149 Physical Activity Minutes), or (c) completely inactive (no Physical Activity Minutes) [44]. 

### 2.2. Exercise Intervention

At entry, the 13 participants were assigned randomly to a 12-week aerobic or a resistance training program (7 in aerobic and 6 in resistance training). The total volume and intensity (*i.e.*, moderate) of exercise was matched between conditions to allow for future comparisons of the effectiveness of different forms of exercise training. Patients remained on their medications throughout the exercise training intervention. Individualized exercise prescriptions were created by qualified exercise professionals according to the clinical exercise prescriptions established by Warburton and colleagues [47,48,49] (see below). 

#### 2.2.1. Aerobic Exercise Intervention

Individuals randomized to the aerobic exercise intervention were asked to participate in a 12-week supervised exercise program consisting of 30 min of moderate-to-vigorous intensity exercise (see Table 2), three times per week on three different exercise modalities (cycle ergometry, treadmill, and elliptical training). Progression was based on heart rate changes (approximately 5% per week). All exercise programs began with a standardized 10 min warm-up and cool-down period involving light aerobic exercise (30% of heart rate reserve) and stretching, respectively. The progress of each participant was reviewed by qualified exercise professionals on a weekly basis. Each training day took approximately 1 h to complete. 

**Table 2:** Example aerobic prescription for a person living with schizophrenia. Adapted from [47].

Program Stage	Week	Frequency (day/week)	Intensity				Duration (min)
			%HRR	%HRmax	RPE	Breathing Rate	
**Initial Stage**	1	3	20–39	50–63	1.5–2.5	Slightly Increased	15–30
Light to moderate intensity aerobic exercise.	2	3	40–50	64–70	3–4		15–30
	3	3	40–50	64–70	3–4	Noticeably Increased	30
	4	3	50–60	70–77	3–5		30
**Improvement**	5–7	3	50–60	70–77	3–5	Noticeably Increased	30
Exercise intensity and duration increase with fitness. Try to achieve health and fitness goals.	8–10	3	60–70	77–84	3–6		30
	11–13	3	60–70	77–84	3–6		30
	14–16	3	65–75	80–87	5–7		30
	17–20	3	65–75	80–87	5–7	More Difficulty Talking while Exercising	30
	21–24	3	70–80	84–90	5–8		30
**Maintenance**	25–28	3	70–80	84–90	5–8	More Difficulty Talking while Exercising	30–45
Try to maintain health-related fitness.							

%HRmax—percentage of maximum heart rate; %HRR—percentage of heart rate reserve; min—minutes; RPE—Rating of Perceived Exertion (10 Scale).

#### 2.2.2. Resistance Training Intervention

Individuals randomized to the resistance exercise intervention participated in a 12-week supervised exercise program consisting of 30 min of moderate-intensity resistance exercise (50%–70% of one-repetition maximum) using the major muscle groups, three times per week. Participants were required to complete two sets of 10–15 (to moderate fatigue) repetitions using 8–10 different exercises from the major muscle groups (*i.e.*, chest press, shoulder press, latissimus dorsi pulldown, leg press, leg extension, leg curl, triceps pushdown, and arm curls) (Table 3). Various functional exercises were also used. The total volume of activity was estimated to match what would have been attained if assigned to the aerobic exercise intervention. 

Small progressions in weight occurred according to the individual participant’s symptoms and musculoskeletal adaptations (*i.e.*, based on changes in the number of repetitions at a given weight (approximately 5% per week)). All exercise programs began with a standardized 10 min warm-up and cool-down period involving light aerobic exercise (30% of heart rate reserve) and stretching, respectively. The progress of each participant was reviewed by the qualified exercise professionals on a weekly basis. Each training day took approximately 1 h to complete.

**Table 3:** Example resistance training program for persons living with schizophrenia. Adapted from [49].

Program Stage	Week	Frequency (day/week)	Intensity	Sets	Muscle Groups
**Familiarization**	1–4	1–2	Light to Moderate 15–20 repetitions RPE = 2–3	1	**8 Major Muscle Groups**
Light intensity upper & lower body resistance exercises Flexibility exercises					At least one exercise per major muscle group (as tolerated) Recommended to use large muscle groups first Alternate between upper and lower body exercises to facilitate recovery
**Goal Specific**	5–24	2–3	Moderate 10–15 repetitions * RPE = 3–5	1–2	
Moderate intensity upper & lower body resistance exercises Flexibility exercises					
**Motor Function and Health-Related Physical Fitness Maintenance**	>24	2–3	Moderate 10–15 repetitions * RPE = 3–5	2–3	

Repetition refers to the completion of a single exercise. Set refers to a number of repetitions performed consecutively until reaching fatigue. Fatigue refers to when the participant is unable to complete the exercise in a correct manner. For each set of exercise, the range of repetitions (e.g., 10–15) refers to the participant being able to complete this number of repetitions before reaching fatigue. The resistance will need to be adjusted accordingly if a participant is able to lift more than recommended repetitions. * More physically fit individuals may wish to complete 8–12 repetitions (representing a higher absolute workload). Participants should complete each repetition in the full range of motion (without pain) with a moderate speed of movement (approximately 6 sec per repetition (2 and 4 s, respectively, for the concentric eccentric phases of the lift)).

### 2.3. Statistical Analyses

For this pilot investigation, we evaluated the changes in the primary measures of interest using a two-way mixed model ANOVA training group (between) and time (within factor). The level of significance was set *a priori* at *p* < 0.05. All data are presented as the mean ± SD. We also calculated clinically relevant changes in the primary outcome measures. Specifically, we compared the change seen as a result of the intervention in the context of a minimally clinically relevant difference as determined by the literature and/or independent clinician judgement. The “clinically relevant difference” is the point at which clinicians and researchers deem the change to be of clinical importance for the patient. Specifically, based on our systematic reviews of the literature and Canadian standards a clinically relevant change in VO_2peak_ would be 0.5 METs (or 1.75 mL•kg^−1^•min^−1^) translating directly to a reduced risk for premature mortality [27,48,50]. Similarly, a 2 mmHg reduction in blood pressure would be of clinical relevance owing to the marked reduction in the risk for stroke and cardiovascular disease with every 2 mmHg reduction in systolic blood pressure [51]. A short-term clinically relevant change in waist circumference has been recently estimated to approximate 5% (or 3.0–6.8 cm) [52]. The National Institutes of Health also suggested that a change in waist circumference of 4 cm is of clinical relevance [53].

## 3. Results

Clinical and demographic data are presented in Table 4. The participants were largely Caucasian with no college or university education. All were not working the year prior to being tested. All patients were currently taking at least one anti-psychotic medication. 

**Table 4:** Clinical and Demographic Data (mean ± SD).

Variable		Baseline Mean SD
Age		30.9 ± 7.2
PANSS		99.2 ± 11.7
Gender		
	Male	7
	Female	6
Ethnicity (N)		
	Caucasian	9
	Aboriginal	3
	Haitian	1
Education (years)		10.8 ± 1.4
WTAR Full Scale Predicted IQ		94.6 ± 6.8
KBIT		81.3 ± 15.0
ROCFT		
	Immediate (T score)	20.3 ± 7.5
	Delayed (T score)	19.7 ± 7.7
HVLT-R		
	Immediate	29.2 ± 11.2
	Delayed	25.1 ± 7.2
	Trails A (raw score)	43.1 ± 24.0
	Trails B (raw score)	142.3 ± 106.5
	Digit Span (Standardized score)	22.8 ± 4.6
	COWA	28.4 ± 7.5
* Antipsychotic Medications		
	Clozapine	246.7 mg•day^−1^
	Olanzapine	11.6 mg•day^−1^
	Aripriprazole	15.0 mg•day^−1^
	Risperidone	LA 25 mg Q2w
	Paliperidone	100 mg•day^−1^
	Flupenthixol	LA inj. 100 mg
	Loxapine	80 mg•day^−1^

PANSS—Positive and Negative Syndromes Scale; WTAR—Weschler Test of Adult Reading; KBIT—Kaufman Brief Intelligence Test; ROCFT—Rey-Osterreith Complex Figure Test; HVLT-R—Hopkins Verbal Learning Test Revised; COWA—Controlled Oral Word Association test. * 6 out of 10 patients were receiving 2 or more concurrent antipsychotic medications.

With respect to the participants’ anthropometry and body composition, 69% were overweight or obese, with 46% being obese (Table 5). When considering waist circumference, 54% of the participants exhibited abdominal obesity (using the standards of the International Diabetes Federation) [54,55]. In the entire group, 62% (5 M and 3 F) had skinfold levels that are associated with a high risk. Approximately 92% had skinfold levels outside of the range believed to associated with optimal health benefits according to Gledhill and Jamnik [38]. 

When considering body mass index and waist circumference together, 69% (9 of 13) of the participants would be considered to be at an increased risk for the development of cardiovascular disease, diabetes, and hypertension (despite the relatively young age of the participants). Six out of 13 (46%) of the participants were classified as being at a very high risk for these chronic conditions. 

According to the Godin Shephard Leisure Time Questionnaire approximately 46% of the patients were exercising below Canada’s physical activity guidelines for apparently healthy adults and below a level that is thought to be associated with substantial health benefits [45]. Approximately 30.8% were completely inactive (*i.e.*, participated in no moderate-to-vigorous activity on a weekly basis). The mean score for the Godin Shephard Leisure Time questionnaire was 28.8 ± 17.3 units with a large range (3–54). The self-reported minutes of moderate-to-vigorous physical activity was 126.9 ± 116.1 min with a large range (0–315).

**Figure 1:**
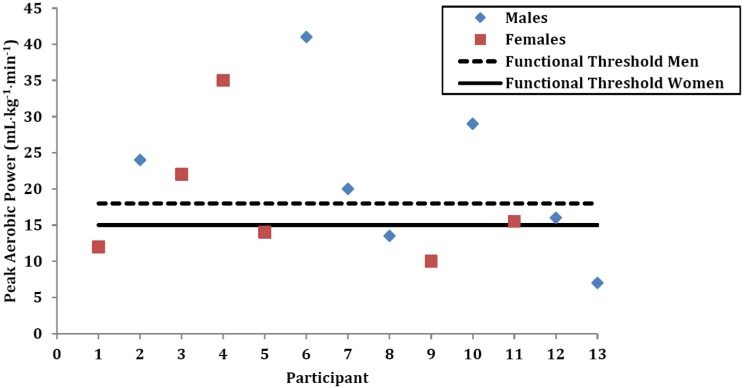
Aerobic fitness of the 13 participants at baseline.

The directly assessed aerobic fitness of the participants averaged 20.5 ± 11.1 mL•kg^−1^•min^−1^ (M: 21.5 ± 11.2 *vs.* F: 19.3 ± 11.9 mL•kg^−1^•min^−1^; Figure 1). The participants’ VO_2peak_ values were well below age- and sex-predicted norms (*i.e.*, 56.8% ± 24.8% of predicted) for apparently healthy individuals. Eleven of the 13 (84.6%; 6 M and 5 F) had a VO_2peak_ that was below predicted values. Six of the participants (46.2%; 3 M and 3 F) exhibited aerobic capacities that are considered to be below what is required for full independent living (*i.e.*, the ability to carry out activities of daily living independently) (Figure 1).

**Table 5:** Anthropometric characteristics of 13 participants at baseline.

	Gender	Height (cm)	Weight (kg)	Body Mass Index (kg•m^−2^)	Overweight and Obesity Class	Waist Circumference (cm)	Waist Circumference Classification	Sum of Five Skinfolds (mm)	Overall Risk Category
	F	165.0	85.0	31.2	Obesity Class I	113.5	Abdominal Obesity	175.0	Very High
	F	156.4	58.4	23.9	Normal	97.0	Abdominal Obesity	87.0	Normal
	F	165.3	51.0	18.7	Normal	76.0	Normal	64.0	Normal
	F	172.3	64.7	21.8	Normal	69.0	Normal	96.0	Normal
	F	165.7	91.2	33.2	Obesity Class I	120.0	Abdominal Obesity	147.0	Very High
	F	164.0	106.0	39.4	Obesity Class II	121.0	Abdominal Obesity	210.9	Very High
**Mean**	**-**	164.8 *	**76.1**	**28.0**	**-**	**99.4**	**-**	**130.0**	**-**
**SD**	**-**	**5.1**	**21.3**	**7.9**	**-**	**22.7**	**-**	**56.9**	**-**
	M	185.5	83.0	24.1	Normal	95.0	Normal	68.5	Normal
	M	187.5	97.5	27.7	Overweight	97.5	Normal	90.5	Increased Risk
	M	167.7	71.9	25.6	Overweight	90.5	Normal	70.3	Increased Risk
	M	177.0	104.5	33.4	Obesity Class I	114.0	Abdominal Obesity	155.4	Very High Risk
	M	179.6	113.7	35.2	Obesity Class II	99.0	Normal	122.5	Very High Risk
	M	180.0	96.6	29.8	Overweight	103.5	Abdominal Obesity	133.0	High Risk
	M	178.5	106.4	33.4	Obesity Class I	112.0	Abdominal Obesity	180.0	Very High Risk
**Mean**	**-**	**179.4**	**96.2**	**29.9**	**-**	**101.6**	**-**	**117.2**	**-**
**SD**	**-**	**6.4**	**14.4**	**4.3**	**-**	**8.7**	**-**	**42.7**	**-**
**Group Mean**	**-**	**172.7**	**86.9**	**29.0**	**-**	**100.6**	**-**	123.1	**-**
**SD**	**-**	**9.4**	**20.1**	**6.0**	**-**	**15.9**	**-**	48.1	**-**

Note: The waist circumference classification is based on the International Diabetes Federation consensus recommendations for Europeans (*i.e.*, abdominal obesity males ≥94 cm, females ≥80 cm). The Overall Risk Category relates to the relative risk for cardiovascular disease, diabetes, and hypertension taking into consideration body mass index and waist circumference (according to the guidelines of the National Heart, Lung & Blood Institute). <18.5 kg•m^−2^, Underweight; 18.5–24.9 kg•m^−2^, Normal; 25.0–29.9 kg•m^−2^, Overweight; 30.0–34.9 kg•m^−2^, Obesity Class I; ≥35.0 kg•m^−2^, Obesity Class II. * Significant difference between sexes.

There was an increased risk with respect to resting blood pressure and heart rate. The mean systolic blood pressure and diastolic blood pressures for the group were 117 ± 16 mmHg and 79 ± 14 mmHg, respectively. This average blood pressure is within the normal clinical range; however, several patients exhibited high blood pressure (according to the American Heart Association guidelines). In the entire group, one participant (8%) exhibited Stage I Hypertension, one had Stage II Hypertension (8%), and five others had Pre-Hypertension (38%). Therefore, seven out of 13 (54%) of the participants had elevated blood pressure. No participant had evidence of hypotension (*i.e.*, systolic blood pressure of 90 mmHg or lower and/or diastolic blood pressure of 60 mmHg or lower). In the males, 71% had elevated blood pressure placing them at increased risk for the development of heart disease. In the females, 33% had higher blood pressure. The resting heart rate was 93.2 ± 17.1 bpm. In our study, 85% of the participants had a heart rate above 90 bpm placing them at an increased risk for cardiovascular disease. 

The lipid profile of our participants was generally within normal limits (cholesterol = 3.68 ± 0.52 mmol•L^−1^; triglycerides = 1.37 ± 0.50 mmol•L^−1^; low-density lipoprotein = 1.80 ± 0.55 mmol•L^−1^; total cholesterol to high-density lipoprotein ratio = 3.48 ± 1.34). However, the average high-density lipoprotein (1.28 ± 0.71 mmol•L^−1^) was below recommended. When examining the individual data, 75% of the participants had high-density lipoprotein values below recommended. Of these individuals, approximately 60% were considered to be at high risk owing to low levels of high-density lipoproteins.

The exercise training program was well tolerated by the participants enrolled in the study. There were no incidences of exercise-related adverse events during testing and/or training (conducted/supervised by qualified exercise professionals). Out of the first 13 participants, four were withdrawn from the study (one for prohibited drug usage, one for non-adherence to appointments, and two were released to their local region and therefore not able to continue in the Vancouver-based program), while nine participants completed the 12-week trial. Thus, 69% of the participants completed the entire training program. However, all participants (irrespective of dropout) remarked on the value of engaging in exercise training, and how the exercise intervention improved their overall well-being. Importantly, the exercise adherence rate (*i.e.*, the participation in recommended exercise prescription) was 81% ± 21% (Range 48%–100%). 

**Figure 2:**
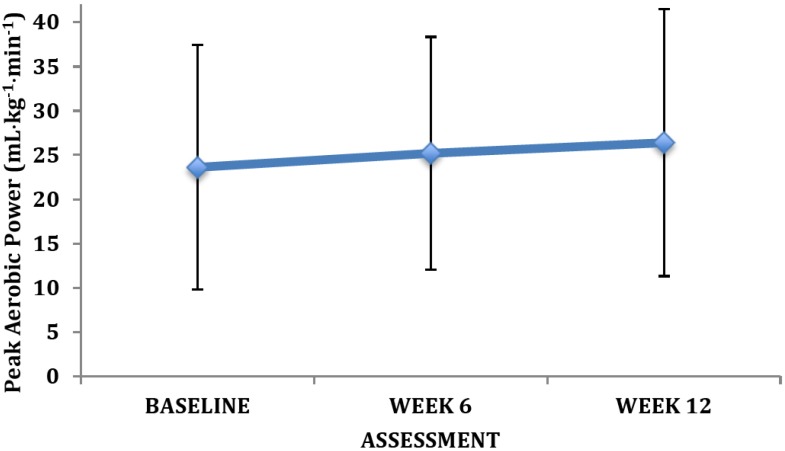
Changes in peak aerobic power with exercise intervention (mean ± SD).

**Figure 3:**
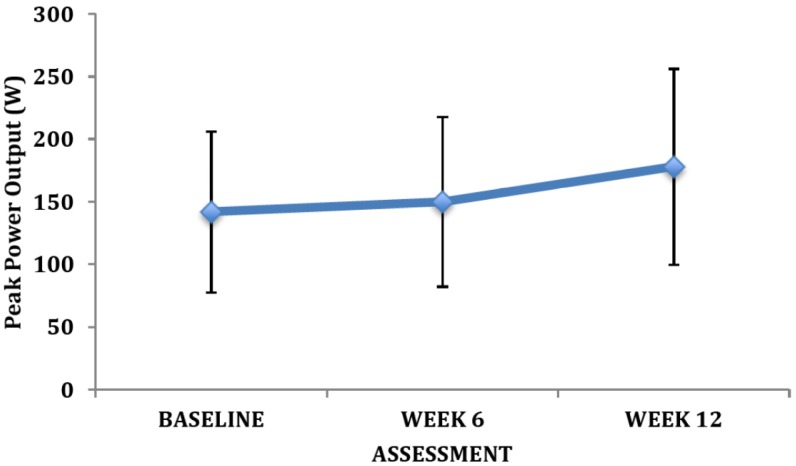
Changes in peak power with exercise intervention (mean ± SD).

**Figure 4:**
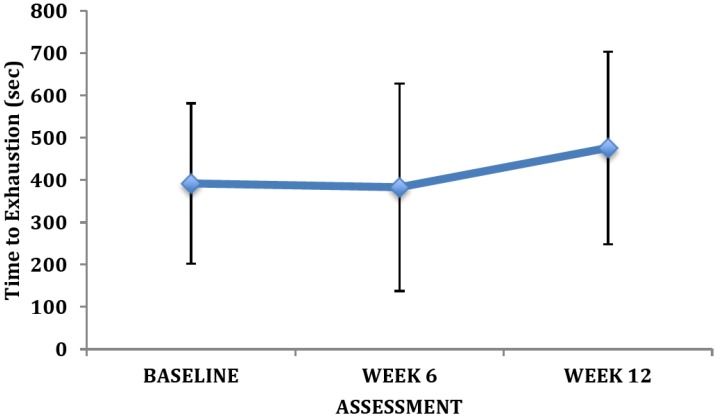
Changes in time to exhaustion with training (mean ± SD).

Our preliminary findings revealed clinically relevant changes in many of the primary outcome measures. Owing to the small sample size there were limited statistically significant differences between training groups and across training. However, the changes were of great clinical relevance. For instance, there were clear improvements in the exercise tolerance measures after training (Figure 2, Figure 3, Figure 4) as reflected by VO_2peak_, peak power output, and time to exhaustion. There was no change in the peak heart rate. The average change in VO_2peak_ observed at week 12 was 2.8 ± 3.4 mL•kg^−1^•min^−1^ (12%).

By design there was a marked (statistically significant) increase in the physical activity of the participants and this was tracked relatively well by the self-report data. In those individuals that completed the trial there was a significant increase in self-reported physical activity (2.23-fold increase in the amount of minutes of moderate-to-vigorous activities and a 4.63-fold increase in the Godin Shephard Leisure Time score). Of note, one participant reported engaging in no moderate-to-vigorous physical activity at either time period, despite having engaged in approximately 3 h of physical activity as part of this study (as monitored by our research staff). 

The training intervention resulted in a slight reduction (approximately 3 kg by week 12) in body mass (Pre = 74.0 ± 18.0, Week 6 = 73.9 ± 14.2, Week 12 = 71.3 ± 14.2 kg) in the participants that completed the exercise intervention. There was also a clinically relevant reduction in waist circumference with the intervention (Pre = 89.3 ± 17.6, Week 6 = 82.9 ± 13.3, Week 12 = 82.7 ± 9.8 cm). There was no marked change in the blood lipid parameters with the intervention. 

There was a clinically relevant change in resting systolic blood pressure with the intervention in the participants that completed the study (Pre = 108 ± 7, Week 6 = 106 ± 11, Week 12 = 104 ± 3 mmHg). There was also a slight reduction in resting diastolic blood pressure (Pre = 75 ± 9, Week 6 = 74 ± 10, Week 12 = 72 ± 7 mmHg).

It should be highlighted that our referring psychiatrists reported anecdotally that the symptom severity of the patients enrolled in the program was improving incrementally over the time. This was supported by a clinically relevant improvement (15.8% reduction) in symptom severity (as assessed by the Positive and Negative Syndromes Scale) in the participants that completed the intervention. 

## 4. Discussion

In the present investigation, we outline our experiences with clinical exercise rehabilitation in persons living with schizophrenia. We specifically outline the potential benefits of exercise training, and our experiences and challenges with providing formalized exercise training with this population. Through this research we were able to report several key findings: (1) persons living with schizophrenia have blood pressure, heart rate, aerobic fitness, HDL levels, and body composition levels that place them at a markedly increased risk for the development of functional limitations (e.g., the inability to complete activities of daily living), cardio-metabolic disease, and/or premature mortality, (2) many persons living with schizophrenia generally exhibit physical activity behaviours that are associated with significant health risk, (3) relatively high levels of adherence to a 12-week exercise rehabilitation program can been achieved through innovative and dynamic approaches, (4) an individualized exercise prescription is required for those living with schizophrenia; generic physical activity guidelines do not appear to be appropriate for this population, and (5) exercise training appears to improve the health and well-being of persons living with schizophrenia including improvements in symptom severity. 

It has been estimated that persons with schizophrenia will die 10–25 years earlier than the general population [20]. Preventable diseases (such as cardiovascular disease and diabetes) explain a large portion of the increased risk for premature mortality [20]. Alarmingly, unlike other chronic conditions wherein the survival rates have increased over recent decades (such as cancer), the gap in premature mortality for persons living with major mental illness has actually increased since the 1990’s [15]. This marked increase over a short period of time is of great concern placing a significant burden on the patient, his/her family, and society as a whole.

Addressing the risk for cardio-metabolic disease and premature mortality in persons with major mental illnesses has important implications for the illness course of these patients. The brain is particularly susceptible to obesity-related damage [56]. Obese individuals demonstrate consistently decreased total brain volumes (in particular reduced gray matter) [57,58,59,60]. Moreover, animal models demonstrate a relationship between experimentally induced weight gain and reduced brain volume [61]. Retrospective investigations have demonstrated increased suicide attempts and a greater frequency of manic and depressive events in obese patients with major mental illness [62,63,64,65]. Obese patients with major mental illness also have more frequent depressive events and shorter periods of euthymia (non-depressed mood) [66]. Members of our research team have recently demonstrated that there is a direct relationship between elevated BMI and reduced brain volumes in obese patients with bipolar I disorder [56]. This research demonstrated the potential biological mechanism between the relationship between obesity and more severe illness course in persons living with major mental illness. 

In our investigation, the majority (69%) of our participants were overweight or obese, with 54% demonstrating abdominal obesity. Remarkably, 46% of our patients were obese. The prevalence of obesity in our participants is well above that reported in the literature for the general population [67]. Overweight and obese individuals are at an increased risk for premature mortality and the development of cardiovascular disease and diabetes in comparison to normal weight individuals [48,68]. Our findings are consistent with other trials revealing a marked prevalence of overweight and obesity in persons living with schizophrenia [13]. In comparison, approximately 59% of Canadian adults (via self-report) are overweight or obese, with 2% underweight, and 39% having a desirable weight [69]. As such, the prevalence of overweight and obesity in our patient cohort is significantly above population-based norms. Importantly, our measures were directly taken by a highly qualified health professional and as such do not reflect bias related to self-report. Collectively, our findings indicate that approximately 60%–70% of the participants (both men and women) were at an increased risk for the development of the metabolic syndrome, diabetes, and cardiovascular disease based on their body weight and composition. As outlined above, obese patients with schizophrenia may be susceptible to obesity-related brain damage, and manic and depressive events. As such, addressing the issue of overweight and obesity in schizophrenia is of great importance. 

In the present investigation, 16% of the participants were found to have high blood pressure (>140/90), and another 38% were pre-hypertensive. In comparison, the global prevalence of elevated blood pressure is estimated at 26% [70]. Approximately 5 million adult Canadians (19% of the population) live with high blood pressure and another 20% of live with pre-hypertension [71]. Therefore, in our cohort we observed an approximate 2-fold prevalence of pre-hypertension *versus* the general population. The adverse effects on blood pressure may be even greater considering the established effects of anti-psychotic medications on blood pressure, specifically marked hypotension. For instance, hypotension is actually a well established side-effect of newer generation anti-psychotic medications (such as clozapine and olanzapine) [72]. In our study 54% of the participants had elevated blood pressure, and not hypotension as would be expected from prolonged usage of second-generation anti-psychotic medications. There is a well-established association between hypertension and cardiovascular disease-related morbidity and mortality [73]. Blood pressure is related linearly to cardiovascular disease risk, with increasing risk moving from normotension to pre-hypertension to hypertension [74]. Moreover, hypertension is also associated with various other chronic conditions (such as chronic heart failure, diabetes [75] and stroke [74]). The early detection and treatment of hypertension is of paramount importance to the attenuation of the risk and costs associated with hypertension and related co-morbidities. Based on our blood pressure findings, we have clear evidence for an increased risk for cardiovascular disease warranting attention. This is particularly important considering the anticipated blood pressure lowering effects of anti-psychotic medications and the prevalence of tobacco smoking in our patients. 

Growing evidence demonstrates resting heart rate provides important insight into the risk for chronic disease and premature death [40,76,77]. This association has been shown in apparently healthy individuals and those with chronic conditions. This relationship is graded such that lower heart rates carry a lower risk for chronic disease. Moreover, the risk of an elevated heart rate appears to be even greater in participants with a history of smoking [76]. In our trial, eight out of the 13 (61.5%) participants were currently smoking with an average intake rate of 5 ± 4 cigarettes per day. Objectively, a heart rate of 90 bpm has been associated with at least a doubling of the risk for cardiovascular disease [40]. Approximately 85% of our patients living with schizophrenia had a heart rate above 90 bpm further reinforcing the increased risk seen in this population. 

Blood lipid levels are well known predictors of the risk for developing diseases of the cardiovascular system. Persons living with schizophrenia may be particularly susceptible to lipid abnormalities and related cardiovascular disease owing (in part) to lifestyle behaviours (such as physical inactivity and poor nutrition) and the metabolic side effects of anti-psychotic medications (such as risperidone, clozapine, and olanzapine) [15,78]. In our study, the lipid profile of our patients was generally within normal limits with the exception of high-density lipoprotein that was below recommended levels. Approximately 60% of the patients in our study had below recommended HDL levels representing an increased risk for cardiovascular events. 

There is substantial evidence that physical inactivity is associated with a marked increase in the risk for the metabolic syndrome, type 2 diabetes, and related comorbidities (in particular cardiovascular disease). Physical inactivity has been linked directly to 16% of type 2 diabetes and 15% of heart disease in British Columbia [79] (with similar estimates across Canada [80,81]). Fortunately, there is clear evidence that routine physical activity is an effective primary and secondary preventative strategy against the development of cardio-metabolic disease [48,81]. Regular physical activity can increase weight loss and improve body composition [27,48,81]. Routine physical activity can also significantly improve glucose tolerance and insulin sensitivity independent of changes in weight loss and body composition [82,83]. 

Exercise prescription for patients with (or at risk for) cardio-metabolic disease is clearly beneficial for improving glucose homeostasis and quality of life [48,81]. It is clearly important to prevent and treat the increased cardio-metabolic risk in schizophrenia [84]. The majority of persons living with major mental illness report being interested in becoming more physically active [85], and understand the health benefits and enjoyment of becoming more physically active [86]. However, the uptake of physical activity guidelines is extremely difficult in persons living with mental illness [87,88]. Other studies have revealed that only 29%–35% of persons living with severe mental illness are able to achieve recommended physical activity levels [87,88], with 15%–25% engaging in no physical activity [18]. Those with limited social contact and women appear to be at the greatest risk for being physically inactive [89]. In our study, using self-reported physical activity levels 46% of the patients were exercising below Canada’s physical activity guidelines for apparently healthy adults and below a level that is thought to be associated with substantial health benefits [45]. This level is below that seen in the general population (*i.e.*, approximately 54%–60%) using this questionnaire [90]. Importantly, 30.8% were completely inactive (*i.e.*, participated in no moderate-to-vigorous activity on a weekly basis). It should be highlighted that the Godin Shephard Leisure time questionnaire has been extensively validated, and used with various clinical populations (including persons living with schizophrenia [91]). However, our findings indicate that further research is warranted to determine the accuracy of self-reported physical activity in comparison to direct measures of physical activity in schizophrenia patients. 

The aerobic capacities were well below predicted (*i.e.*, 56.8% of predicted) with the vast majority of the patients (84.6%) having VO_2peak_ values below predicted. This is consistent with findings from other high-risk clinical populations such as severe heart failure [92]. In fact, a significant proportion of our participants (46.2%) exhibited aerobic capacity values near the threshold for independent living [92]. Importantly, the health-related physical fitness values observed in our trial in a relatively young population (Age = 31 ± 7 years) were similar to what we observe in participants 60 years of age or older. As such, many individuals were demonstrating health-related physical fitness levels that are associated with premature aging. This level of health-related physical fitness places these individuals at marked risk for the development of cardio-metabolic disease and premature mortality, and in jeopardy for losing the ability to carry out activities of daily living (*i.e.*, to live independently) [27]. 

Consistent with previous literature [27], there was a disconnect between self-reported physical activity and directly assessed physical activity and/or fitness. The direct measurement of aerobic capacity demonstrated a marked impairment and increased risk in the majority of participants; however, 54% of the participants reported engaging in recommended levels of physical activity. The higher physical activity levels in the face of marked impairments in aerobic capacity supports previous concerns about self-report bias in physical activity questionnaires (particularly in clinical populations) [27]. Our findings further emphasize the importance of direct assessments of aerobic capacity when assessing the health status of persons living with major mental illness. 

Exercise training for persons living with schizophrenia has recently been advocated as potential potent non-pharmacological intervention. Exercise is an important therapeutic intervention for schizophrenia owing to the potential to address the symptoms of the condition, improve cognitive function, and reduce the risk for premature mortality and the development of various secondary chronic conditions (such as diabetes and heart disease). Unfortunately, previous research has indicated that the majority of patients with major mental illness that engage in traditional physical activity programs withdraw shortly after starting. Patients often report limited confidence in their ability to exercise and low levels of support from their social network and health professionals [93]. As such, better exercise intervention strategies are warranted [94,95]. These strategies require an integrated approach involving the collaboration of the individual patient with qualified exercise professionals [96,97], neurocognitive specialists, physicians, and other allied health professionals. The application of generic physical activity guidelines to their treatment is not recommended. In our trial, we encountered challenges with exercise adherence over the 12 weeks of the study. However, the majority of these challenges were relating to issues unrelated to the exercise intervention (such as substance usage and the inability to make appointments). In the end, 69% of our participants were able to complete the entire trial (*i.e.*, there was a low dropout rate). It is important to highlight that dropout rates in clinical trials involving persons living with schizophrenia are quite high (irrespective of the intervention). For instance, trials of antipsychotic medications often demonstrate dropout rates of greater than 50% [98]. In our current trial, the reasons for dropout were related largely to the medical management of the patients (*i.e.*, the transfer of patients to another facility) and not their unwillingness or inability to participate in the exercise program. Moreover, the exercise adherence rate was quite good (81% ± 21%). The low dropout and high exercise adherence rate are remarkable reflecting the importance of innovative and dynamic approaches to exercise training in schizophrenia. The majority of the participants were able to accrue health benefits (including reductions in symptom severity) during the research investigation. The benefits of this initiative were reflected directly by the confidence afforded this research with the development of the first of its kind brain health and wellness centre at the University of British Columbia. It is envisioned that this facility and its program of research will affect positively the health and well-being of thousands of Canadians living with severe mental illness. 

We observed several finding that are of clinical and physiological relevance for persons living with schizophrenia. By design we observed a marked increase in the physical activity levels of the patients enrolled in the program. Our intervention involved 90 min weekly of moderate intensity physical activities, with an additional 90 min of light intensity activities (during the warm-up and cool-down periods). This exercise prescription for moderate-to-vigorous exercise is far less than that recommended by international physical activity guidelines for healthy individuals (*i.e.*, 150 min); but consistent with recommendations for other clinical populations [99]. It is also important to highlight, that based on the exercise intensity, the weight of the participants, and estimates for the metabolic requirements of the activities engaged in [100] our intervention equated to an energy expenditure of approximately 1006 ± 229 kcal per week. Modest enhancements in physical activity/fitness in previously inactive individuals can be associated with large improvements in health status [27,48,81]. For instance, a 1000 kcal per week increase in physical activity or 1 MET increase in aerobic fitness has been associated with a 20%–30% reduction in the risk for premature mortality [101]. A half MET increase in aerobic fitness is also thought to be of significant clinical benefit [50]. In our study, the participants exhibited a 2.8 mL•kg^−1^•min^−1^ (12%) change in VO_2peak_ at week 12. Therefore, the early data from our new research centre supports the health benefits of individualized exercise prescription in persons living with schizophrenia. Our findings are consistent with others that have demonstrated the efficacy of exercise training in persons living with major mental illness [102,103].

Other important markers of health status were also affected positively by the exercise intervention. For instance, our patients did not increase their body weight over the 12-week intervention, as generally seen in patients owing to the metabolic side effects of medication. There was a clear trend for an improvement in body mass and abdominal obesity in those that completed the intervention. This is consistent with the findings of other researchers [102,104] demonstrating the potential benefits of exercise training for mitigating the weight gain associated with psychotropic medications. Similarly, systolic blood pressure was reduced after the intervention. The average blood pressure reduction was 4 mmHg. To put this into a health-related context, a 2 mmHg reduction in systolic blood pressure has been associated with a 14% and 9%, respectively, reduction in the risk of stroke and coronary artery disease [51]. 

It is clear that exercise training can improve health-related physical fitness and reduce the risk for premature mortality and other chronic conditions. Moreover, growing evidence supports the potential benefits of exercise training on cognitive function. For instance, a recent investigation (involving the head of our psychiatry program) revealed that three months of aerobic exercise training (cycling) was associated with an increase in hippocampal volume (12%) in patients living with schizophrenia, and that exercise-related changes in hippocampal volume were correlated with improvement in maximal aerobic power [105]. However, another randomized controlled trial from the same team revealed that aerobic exercise training might increase gray matter density (in the right frontal and occipital cortex) in healthy controls, but not necessarily persons living with chronic schizophrenia. A recent systematic review of the literature [28] also revealed that there is preliminary evidence from randomized controlled trials that aerobic and strength exercises (and yoga) may improve short term memory and reduce psychiatric symptoms in schizophrenia. 

In the future, we feel that further research should look at the mechanisms responsible for the improvement in cognitive function and symptom severity seen after exercise training in persons living with schizophrenia. In particular, we feel that further research is required to examine the cognitive benefits related to exercise training in schizophrenia, and the role exercise-induced hippocampal neuroplasticity plays in the improvement in cognition and the reduction of positive symptoms of psychosis. 

Through this pilot investigation, we were able to modify and refine our original clinical exercise prescriptions recommendations for specific usage with persons living with schizophrenia (Table 2, Table 3). A key distinction exists when comparing the exercise prescription for persons living with schizophrenia to that of other chronic conditions. For instance, owing to the marked physiological impairment a conservative approach to the initial stages of exercise is warranted. In our practice, we noticed that these patients often have difficultly engaging in prolonged continuous exercise. As such, shorter duration and/or interval type exercise may be advocated until fitness and exercise tolerance are improved. With respect to resistance training, we have also noticed that a lower resistance and relative intensity are required for each exercise (particularly into the early stages of training). The patients can generally complete one set of approximately eight major muscle groups during a 30 min exercise session. However, additional sets are difficult to complete and the time required is often not conducive to the schedule of the patients (particularly those that are in-patients) (see Table 2). Functional activities appear to be particularly welcome by persons living with schizophrenia. An adequate warm-up and cool-down is also an important component of an effective exercise intervention for schizophrenia. As demonstrated in our findings patients with schizophrenia generally are markedly deconditioned (in comparison to the general population). However, a small portion of the population (in our situation 25%) has normal or near normal aerobic fitness. Therefore, an individualized prescription is required to optimize the health benefits associated with exercise training. The application of generic physical activity guidelines for the treatment of schizophrenia is not recommended. This is consistent with the recommendations of the International Organization of Physical Therapy in Mental Health consensus [106]. 

It is important to highlight that our research team is part of two international collaborations related to the development of clinical exercise prescriptions for chronic medical conditions (*i.e.*, the International Collaboration on Clinical Exercise Prescription and the International Collaboration in Cardiovascular Prevention and Rehabilitation). Through this process we have been able to refine exercise prescriptions for various chronic conditions. This work has formed the foundation for our clinical exercise prescriptions (see [47,48,49]). With this framework, we were able to refine our clinical approach and prescriptions for the specific needs of patients living with schizophrenia.

Another important consideration that differs from traditional rehabilitation is the level of supervision provided to the patients. Unlike other chronic conditions that we commonly work with, we found that our patients with schizophrenia required one-to-one instruction with appropriate medical/safety supervision. Unlike cardiac rehabilitation programming where it is common to have one qualified exercise professional to 4–6 participants, it was clear that schizophrenia patients require a more personalized intervention. Also, our patients preferred to exercise in a small group setting (2–3 participants) with other patients living with schizophrenia. This increases the costs associated with the intervention, but we feel also explains why our patients report such positive feelings regarding the intervention. There was wide spread support for this program ultimately resulting in the development of the first of its kind wellness and research centre dedicated to the non-pharmacological treatment of severe mental illness. The Centre of Excellence in Brain Health and Wellness has been developed specifically to address the needs of those with severe mental illness. 

Practical and financial limitations of employing an exercise intervention within the clinical, in-patient setting exist. We recognize fully the challenges of implementing a program of this nature in the hospital setting. Our multidisciplinary approach involved psychiatrists, qualified exercise professionals, and other allied health professionals working in concert. Our additional costs were related largely to the qualified exercise professionals and research coordinator. However, similar to other clinical interventions, we found that the benefits of the exercise intervention far outweighed the costs/risks for our patients. Our new program has received sufficient support and interest to warrant engaging various other centres and community partners in the development of parallel programs in the Greater Vancouver region. We acknowledge that de-institutionalization is occurring throughout Canada and as such services are increasingly being offered on an outpatient basis. To address this issue we have established key partnerships with organizations and programs dedicated to improving the health and well-being of society. As an example, we have a formal partnership with the Physical Activity Line of BC wherein our patients can receive free daily physical activity and lifestyle behaviour advice [107]. This includes individualized exercise recommendations based on the prescriptions developed as part of this study and our ongoing international collaborations. 

## 5. Conclusion

We have demonstrated a markedly increased risk for cardio-metabolic disease in persons living with schizophrenia including worsened blood pressure, heart rate, aerobic fitness, and body composition. This is consistent with other researchers’ findings [13]. An individualized exercise program has shown early promise leading to the first of its kind dedicated brain health and exercise wellness centre for persons living with schizophrenia. Through our initiatives, we have been able to refine our exercise prescription to address the unique challenges of schizophrenia. This includes developing new and novel means of addressing the limited exercise adherences rates demonstrated in our early work and that of others. We feel that exercise interventions have a remarkable potential to improve cognitive function, functional and physiological status, and overall well-being, while reducing the risk for cardio-metabolic disease in persons living with schizophrenia. 
